# Single-port laparoscopic surgery versus laparotomy for treatment of ovarian cysts ≥ 5 cm in the first and early second trimester of pregnancy: a retrospective comparative study

**DOI:** 10.1186/s12884-026-08841-8

**Published:** 2026-02-24

**Authors:** Haibin Zhang, Lihui Li, Huiyan Wang, Chunna Wei, Zhen Zhang

**Affiliations:** Department of Gynecology, Shijiazhuang Obstetrics and Gynecology Hospital, Shijiazhuang, 050000 P. R. China

**Keywords:** Laparoendoscopic single-site surgery, Laparoscopic assisted surgery, Minimally invasive surgery, Ovary, Single-port

## Abstract

**Objective:**

To investigate the safety and feasibility of single-port access laparoscopic for the surgery treatment of ovarian cysts (maximal diameter ≥ 5 cm) during pregnancy.

**Methods:**

In this retrospective cohort study, 12 pregnant patients who underwent single-port access laparoendoscopic single-site (LESS) surgery (Group 2) for ovarian cysts between January 2021 and July 2022 were compared with 16 pregnant patients who underwent laparotomy during the same period (Group 1). We retrospectively analyzed clinical characteristics and perioperative outcomes, including age, body mass index, cyst size and pathology, operative time, estimated blood loss (EBL), and postoperative length of hospital stay.

**Results:**

No statistically significant differences were observed in baseline characteristics between the two groups. Operative time was significantly shorter in group 2 than in group 1 (56.00 ± 15.06 vs. 95.31 ± 17.82 min, *p* < 0.001). Estimated blood loss was significantly lower in group 2 than in group 1 (12.08 ± 6.20 vs. 33.13 ± 19.57mL, *p* < 0.001). Mean hospital stay was shorter in group 2 (4.00 ± 0.95 vs. 7.88 ± 0.96 days, *p* < 0.001).

**Conclusion:**

In this small retrospective cohort, LESS appeared feasible for selected pregnant patients with ovarian cysts (maximal diameter ≥ 5 cm) and was associated with less blood loss and shorter hospitalization than laparotomy. These findings should be interpreted cautiously given the non-randomized design and limited sample size.

## Introduction

The incidence of ovarian tumors has been reported to range from 0.17% to 5.9% in asymptomatic women and from 7.1% to 12.0% in symptomatic women [1]. Adnexal masses first diagnosed during pregnancy often pose clinical challenges for both obstetricians providing prenatal care and gynecologic surgeons consulted for management. The reported incidence of adnexal masses during pregnancy varies widely, from approximately 1:630 to 1:2020, depending on the population studied and, importantly, on the frequency of routine prenatal ultrasound examinations [2, 3]. Most adnexal masses detected in pregnancy are benign cystic lesions and may resolve spontaneously; however, some persist and may become symptomatic due to hemorrhage, rupture, torsion, or mass effect.

Laparoscopy is an established approach for the management of benign adnexal masses [4]. Given that gestational age is central to surgical decision-making in pregnancy, management is influenced not only by cyst characteristics (e.g., size and persistence) but also by the changing uterine size and limited working space. The optimal surgical approach for larger cysts remains debated. Some authors have suggested that laparotomy may be preferable for adnexal masses larger than 8–10 cm [5, 6]. In recent years, several studies have described perioperative outcomes of single-port access laparoscopic surgery (laparoendoscopic single-site surgery) for adnexal disease [7–10]. However, comparative evidence in pregnant patients—particularly in the first and early second trimester—and for persistent/enlarging ovarian cysts remains limited.

Therefore, the present study was designed to evaluate the feasibility and safety of single-port access laparoscopic surgery for ovarian cysts with a maximal diameter ≥ 5 cm during the first and early second trimester of pregnancy, and to compare perioperative outcomes with laparotomy.

## Methods

Between January 2021 and July 2022, this retrospective cohort study included 16 pregnant patients who underwent laparotomy for large ovarian cysts (Group 1) and 12 pregnant patients who underwent single-port access laparoscopy (LESS) (Group 2) at the Shijiazhuang Obstetrics and Gynecology Hospital. Surgical outcomes, complications, and intraoperative cyst rupture/spillage rates were compared between groups. The Institutional Review Board of our hospital approved the study. Ethics approval No.: 20,240,048.

The choice of surgical approach (single-port [LESS] versus laparotomy) was individualized after shared decision-making. LESS was preferentially offered when (i) preoperative imaging suggested a benign adnexal cyst, (ii) the cyst appeared mobile and amenable to controlled decompression and exteriorization through the umbilicus, (iii) the patient was hemodynamically stable, and (iv) an experienced LESS surgeon and single-port equipment were available. Laparotomy was chosen when additional exposure was anticipated (e.g., very limited working space, poor cyst mobility/suspected adhesions), when urgent access was required based on clinical judgment, when LESS equipment or trained personnel were unavailable, or when the patient preferred open surgery after counseling.

The choice of surgical approach (single-port [LESS] versus laparotomy) was individualized based on surgeon expertise, cyst characteristics, and intraoperative feasibility rather than a predefined allocation protocol.

All patients underwent physical examination, routine laboratory testing, and pelvic ultrasound. When malignancy was suspected, additional evaluation (e.g., abdominal and pelvic magnetic resonance imaging) was performed. Preoperative assessment included prior abdominal surgical history and laboratory studies (complete blood count, routine chemistry, electrolytes, coagulation profile, and serum tumor markers including CA-125), as well as chest radiography and electrocardiography. Because serum CA-125 may be physiologically elevated during pregnancy—particularly in the first trimester—it was interpreted as an adjunct to imaging findings rather than as a stand-alone criterion for malignancy [11, 12]. 

Eligibility criteria were: (1) intrauterine pregnancy with gestational age < 24 weeks; (2) adnexal mass considered benign based on preoperative imaging (no obvious malignant features); (3) maximal cyst diameter ≥ 5 cm on preoperative ultrasound; and (4) suitability for surgery under general anesthesia.

Exclusion criteria included an obviously malignant mass on imaging (e.g., solid components, papillary projections, ascites), markedly elevated serum CA-125 levels (> 500 U/mL), suspicion of severe pelvic adhesions on physical examination, signs of threatened abortion, or a history of abdominal surgery.

### Surgical technique

All procedures were performed by surgeons trained in minimally invasive gynecologic surgery. The choice between oophorectomy and ovarian cystectomy was based on intraoperative findings and the clinical scenario.

**Fig. 1:**
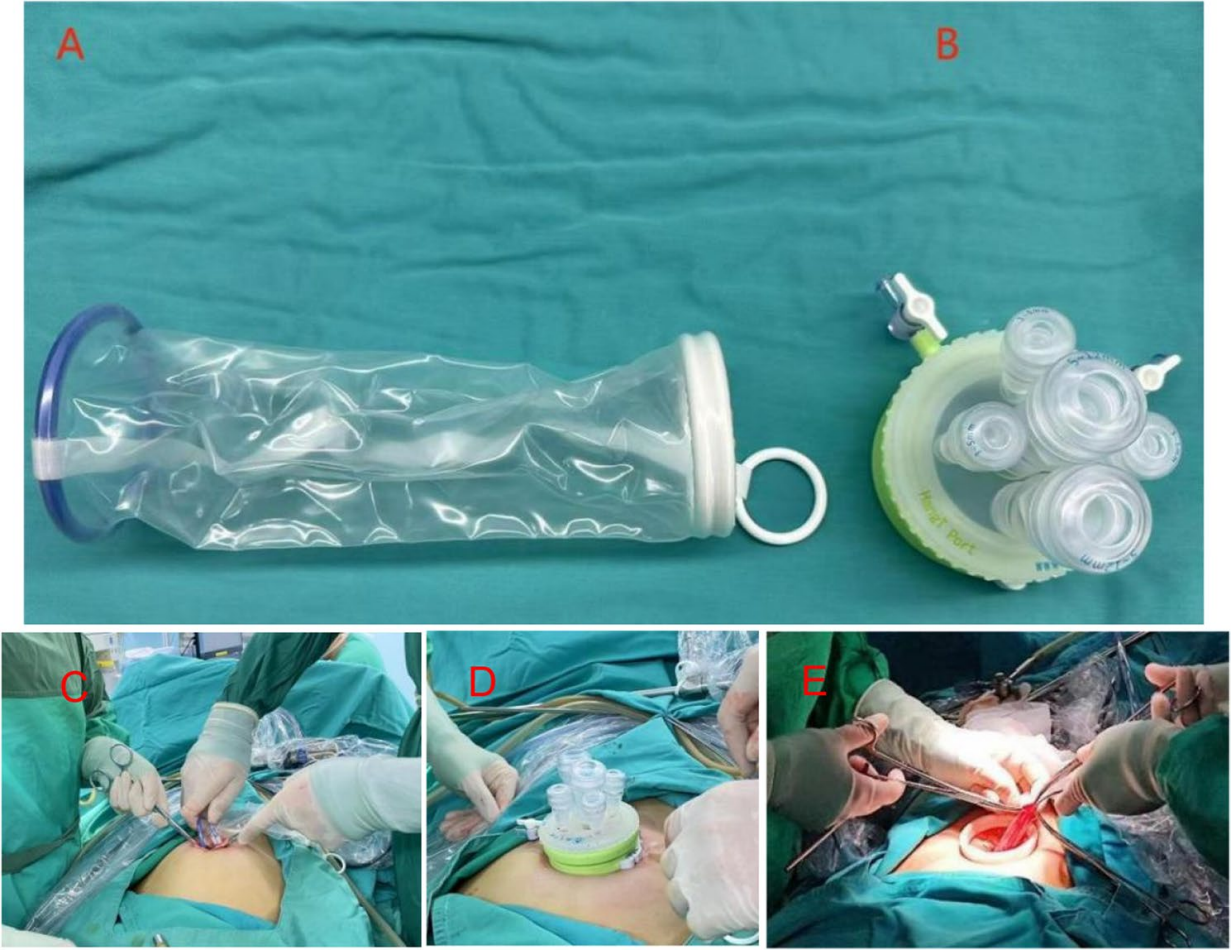
**A** Wound retractor. **B** Port cap.Inserting the laparoendoscopic single-site surgery (LESS) port through a 2–3 cm transverse transumbilical incision. **C** Placement of the wound retractor via the umbilical approach. **D **The LESS part has two 12-mm port and three 5-mm ports. **E **Aspiration of cystic contents and cyst pulled out to the extracorporeal space

Single-port assisted extracorporeal cystectomy was performed using an OctoPort (HangTian KaDi, China), consisting of a 30-mm wound retractor and a detachable port cap with five access channels (two 12-mm ports and three 5-mm ports) (Fig. 1A, B). Under general endotracheal anesthesia, patients were positioned in dorsal lithotomy. A 2.5-cm vertical transumbilical skin incision was made, the subcutaneous tissue was dissected, and the peritoneum was entered. The wound retractor was introduced into the peritoneal cavity (Fig. 1C, D), and the port cap was secured. Pneumoperitoneum was established and maintained at 12 mmHg. A 30° laparoscope was introduced through a 12-mm port to inspect the ovarian cyst, contralateral adnexa, peritoneal surfaces, and omentum. The port cap was then temporarily removed, and the cyst wall was gently exteriorized through the umbilical incision, protected by the wound retractor (Fig. 1E). The cyst was punctured with a needle suction device, and cyst fluid was aspirated in a controlled manner. Extracorporeal cystectomy was performed using blunt and sharp dissection, and the ovarian cortex was reconstructed with continuous absorbable 2 − 0 sutures. The ovary was returned to the peritoneal cavity, the port cap was reattached, and pneumoperitoneum was re-established for final laparoscopic inspection and irrigation. After desufflation, the instruments and wound protector were removed, and the umbilical incision was closed; the procedure was then completed.

For laparotomy, a Pfannenstiel incision was preferred when feasible; an infraumbilical midline incision was used when additional exposure was required based on cyst size or gestational anatomy. Uterine manipulation was minimized. Ovarian cystectomy or oophorectomy was performed according to intraoperative findings, with meticulous hemostasis. Cyst rupture was avoided whenever possible; if rupture occurred, cyst contents were suctioned promptly and the operative field was irrigated thoroughly. The abdominal wall was closed in layers.

Operative time was defined as the time from the first skin incision to the final stitch. Estimated blood loss was assessed by measuring aspirated blood, weighing swabs, and estimating the amount of blood on drapes. Febrile morbidity was defined as a temperature > 38 °C on two occasions 24 h apart. Parameters analyzed included age, body mass index, largest cyst diameter, operative time, blood loss, length of hospital stay, and complications.

Obstetric and neonatal outcomes were obtained from delivery and neonatal records, including gestational age at delivery, pregnancy outcome, mode of delivery, and the primary indication for caesarean section. Neonatal outcomes were summarized among live births and included birthweight, 5-minute Apgar score, and NICU admission.

### Perioperative obstetric management

Perioperative management was coordinated with the obstetric team. All included pregnancies were previable (< 24 gestational weeks). In accordance with evidence-based guidance on laparoscopy during pregnancy, routine intraoperative fetal heart rate monitoring was not performed. Fetal status was reassessed postoperatively using bedside ultrasound, including Doppler/color Doppler as clinically indicated. Prophylactic tocolysis was not routinely administered. Following SAGES recommendations, rescue tocolysis was considered only when signs or symptoms suggestive of preterm labor were suspected or documented perioperatively, at the discretion of the obstetric team.

### Statistical analysis

Statistical analysis was performed using SPSS version 25.0 (IBM, Armonk, NY, USA). Continuous variables were assessed for normality using the Shapiro–Wilk test. Normally distributed data are presented as mean ± standard deviation (SD) and were compared using Student’s t-test; non-normally distributed data were compared using the Mann–Whitney U test. Categorical variables are presented as number (percentage) and were compared using the χ² test or Fisher’s exact test when expected cell counts were < 5. All tests were two-sided, and *p* < 0.05 was considered statistically significant. In addition to p-values, we report effect sizes to aid interpretation: for continuous outcomes, mean differences (MD) with 95% confidence intervals (CI); for categorical outcomes, risk differences (RD) and Fisher’s exact test when appropriate. Given the limited sample size, we did not perform multivariable regression modeling; instead, we emphasize transparent reporting of key baseline factors and effect sizes with 95% CIs.

## Results

A total of 28 patients were enrolled in the study. Table 1 summarizes patient demographics and baseline characteristics. The mean age and body mass index were 28.19 years and 23.59 kg/m^2 in Group 1, and 28.17 years and 24.91 kg/m^2 in Group 2, respectively; no statistically significant differences were observed between groups. Pathology in Group 1 included three mature cystic teratomas (18%), nine mucinous cystadenomas (56%), two serous cystadenomas (13%), and two endometriomas (13%). Pathology in Group 2 included four mature cystic teratomas (33%), five mucinous cystadenomas (42%), two serous cystadenomas (17%), and one endometrioma (8%) (Table 1). Serum CA-125 was measured preoperatively in all patients; no patient exceeded the predefined exclusion threshold (> 500 U/mL).

**Table 1:** Patients and tumor characteristics

Patients and tumor characteristcs			
Variable	Group1(*n* = 16)	Group2(*n* = 12)	p value
Age, yr	28.19 ± 3.75	28.17 ± 3.43	0.98
Body mass index	23.59 ± 2.99	24.91 ± 5.43	0.419
Gestational weeks	16.63(6.19)	15.83(4.57)	0.697
Histologic type			
Mature cystic teratoma	3(18)	4(33)	
Mucinous cystadenoma	9(56)	5(42)	
Serous cystadenoma	2(13)	2(17)	
Endometrioma	2(13)	1(8)	
Largest diameter, cm	12.44(6.13)	10.58(1.83)	0.266
Emergency surgery for ovarian torsion	6(37.50)	4(33.33)	1.000

### Comparison of surgical observation indexes between the two groups

The mean (SD) maximal cyst diameter was 12.44 (6.13) cm in Group 1 and 10.58 (1.83) cm in Group 2 (Table 1). Operative time was significantly shorter in Group 2 than in Group 1 (56.00 ± 15.06 vs. 95.31 ± 17.82 min, *p* < 0.001). Estimated blood loss was significantly lower in Group 2 than in Group 1 (12.08 ± 6.20 vs. 33.13 ± 19.57 mL, *p* < 0.001). Mean postoperative hospital stay was shorter in Group 2 than in Group 1 (4.00 ± 0.95 vs. 7.88 ± 0.96 days, *p* < 0.001) (Table 2).

**Table 2:** Comparison of surgical observation indexes between the two groups

Surgical outcomes, complications			
Variable	Group1(*n* = 16)	Group2(*n* = 12)	p value
Operative time, min	95.31 ± 17.82	56 ± 15.06	< 0.001
Blood loss, ml	33.13 ± 19.57	12.08 ± 6.20	< 0.001
Hospital stay, days	7.88 ± 0.96	4.00 ± 0.95	< 0.001
Time to first flatus, h	39.44 ± 15.13	17.17 ± 5.12	< 0.001
Intraoperative cyst rupture/spillage, n (%)	0 (0.0%)	0 (0.0%)	1.000
Complications			
Fever	3	1	
Wound disruption	0	0	
Urinary tract infection	0	0	
Clostridium difficile colitis	0	0	

Effect sizes were consistent with these findings. Compared with laparotomy, LESS was associated with a shorter operative time (MD -39.31 min; 95% CI -51.51 to -27.11), lower blood loss (MD -21.05 mL; 95% CI -31.26 to -10.84), a shorter hospital stay (MD -3.88 days; 95% CI -4.59 to -3.17), and earlier first flatus (MD -22.27 h; 95% CI -30.23 to -14.31).

### Comparison of postoperative observation indexes between the two groups

No intraoperative complications requiring treatment occurred in either group. Intraoperative cyst rupture/spillage was not observed in either group (0/16 vs. 0/12; *p* = 1.000). Postoperatively, fever occurred in three patients in Group 1 and one patient in Group 2. Time to first flatus was longer in Group 1 than in Group 2 (39.44 ± 15.13 h vs. 17.17 ± 5.12 h, *p* < 0.001) (Table 2). No postoperative incisional hernia occurred in either group. In the single-port group, no conversion to laparotomy and no additional trocar placement were required.

### Comparison of follow-up pregnancy outcomes between the two groups

Follow-up obstetric and neonatal outcomes are summarized in Table 3. Given the marked difference in delivery mode between groups, we additionally reported the primary indications for caesarean section.

**Table 3:** Comparison of follow-up pregnancy outcomes between the two groups

Pregnancy outcome		
Variable	Group 1 (*n* = 16)	Group 2 (*n* = 12 )
Gestational age at delivery, weeks (mean ± SD)*	38.50 ± 1.81	39.00 ± 1.39
Partus maturus	13	11
Premature delivery	2	1
Abortion	1	0
Induced labour	0	0
Delivery mode		
Vaginal delivery	4	10
Caesarean section	11	2
Indications for caesarean section	n (%)	n (%)
Previous caesarean section	3 (27.3%)	0 (0.0%)
Antepartum fever/infection	2 (18.2%)	1 (50.0%)
Nonreassuring fetal status	3 (27.3%)	1 (50.0%)
Placenta previa/placental disorders	0 (0.0%)	0 (0.0%)
Hypertensive disorders	2 (18.2%)	0 (0.0%)
Uterine rupture	1 (9.1%)	0 (0.0%)
Other	0 (0.0%)	0 (0.0%)
Neonatal outcome		
Neonatal asphyxia	0 (0.0%)	0 (0.0%)
Birthweight, g (mean ± SD)	3304 ± 190	3258 ± 286
Apgar score at 5 min (mean ± SD)	9.79 ± 0.58	9.67 ± 0.65
NICU admission, n (%)	0 (0.0%)	0 (0.0%)
Congenital anomaly, n (%)	0 (0.0%)	0 (0.0%)
Perinatal death, n (%)	1 (6.3%)	0 (0.0%)

Neonatal outcomes were summarized among live births. Gestational age at delivery was calculated among deliveries (excluding abortion/miscarriage). Indications for caesarean section represent the primary indication.Serum CA-125 was screened preoperatively as an adjunct to imaging; no patient exceeded the exclusion threshold (> 500 U/mL)

In Group 1, 13 patients had a term delivery and two had a preterm delivery; one miscarriage occurred on postoperative day 4 (gestational age 14 + 5 weeks), and one stillbirth occurred at 33 weeks due to uterine rupture. In Group 2, 11 patients had a term delivery and one had a preterm delivery. The caesarean section rate was higher in Group 1 than in Group 2 (11/15 vs. 2/12, *p* = 0.006). Among live births, birthweight (3304 ± 190 g vs. 3258 ± 286 g, *p* = 0.646) and 5-minute Apgar score (9.79 ± 0.58 vs. 9.67 ± 0.65, *p* = 0.630) were comparable between groups, and no neonate required NICU admission.

## Discussion

In the first trimester of pregnancy, ovarian cysts are often functional and generally resolve without complications. After 16 weeks’ gestation, the prevalence of ovarian cysts has been reported to be between 0.5% and 3.0% [13]. Fagotti et al. reported a prevalence of 0.9% for ovarian cysts beyond 16 weeks’ gestation [14]. In a cross-sectional study of 2245 women scanned at the end of the first trimester, 1.2% of detected cysts persisted beyond 16 weeks and were subsequently removed surgically; no cases of malignancy were identified [1]. In our study, the mean gestational age at surgery was 16.63 weeks in the laparotomy group and 15.83 weeks in the single-port group, which is consistent with previous reports.

In a study of 55, 278 women undergoing termination of pregnancy, two cases of ovarian malignancy were identified [2]. Expectant management of ovarian masses is advocated, at least until the pregnancy is beyond 14 weeks’ gestation. When symptomatic, simple ovarian cysts diagnosed during pregnancy can also be treated with ultrasound-guided cyst aspiration in selected cases [13].

Serum tumor markers such as CA-125 may assist the evaluation of adnexal masses in nonpregnant patients; however, CA-125 can be physiologically elevated during pregnancy, particularly in the first trimester, which limits its specificity. Therefore, we relied primarily on imaging characteristics for malignancy risk assessment and used CA-125 only as an adjunct [11, 12, 15].

Ovarian torsion is an important diagnosis to consider because it warrants urgent intervention. The presence of an ovarian mass markedly increases the risk of torsion [11]. Approximately 10–20% of torsion cases occur during pregnancy, most commonly in the first and early second trimesters [11, 16]. The overall risk of torsion in pregnancy is about 0.1% [16, 17], but it increases to approximately 5–15% in the presence of an ovarian mass [17]. Symptoms in pregnancy are similar to those in nonpregnant women [18]. Examination findings are often more pronounced in torsion than in rupture [19], and patients may be tachycardic, tachypnoeic, and have low oxygen saturations. Pregnant women with torsion may display peritoneal signs less frequently than nonpregnant counterparts [20]. In our study, 10 patients (35.71%) underwent emergency surgery due to ovarian torsion—four in the single-port group and six in the laparotomy group.

Laparoscopic cystectomy for ovarian cysts with a large maximal diameter presents several technical challenges, including limited working space, an increased risk of inadvertent rupture during abdominal entry (e.g., Veress needle or trocar placement), and difficulty with specimen retrieval. In a recent study, both cyst size and cystectomy were positively and significantly associated with inadvertent intraoperative cyst rupture, highlighting the need for meticulous spillage control when managing larger cysts laparoscopically [21]. In pregnancy, adnexal masses measuring ≥ 5 cm are less likely to resolve spontaneously and more often require closer follow-up or surgical evaluation due to symptoms (e.g., pain/torsion risk) and the need to exclude neoplasia based on imaging characteristics, rather than size alone [11, 13, 15, 17, 18, 22]. 

Abdominal entry is a critical step in laparoscopy, and a substantial proportion of major laparoscopic complications occur during entry. In a randomized controlled prospective trial, the median umbilical ligament lift-up technique was compared with Veress needle entry in gynecologic laparoscopic surgery and demonstrated differences in access safety and procedural efficiency, underscoring that the choice of entry method can influence access-related outcomes [22]. In the present study, the single-port approach used a transumbilical open incision with a wound retractor, which may reduce the likelihood of blind needle/trocar-related injury and facilitate controlled decompression and specimen handling for large cysts in early pregnancy.

To overcome the limitations of conventional laparoscopy when treating larger cysts, we adopted a single-port assisted extracorporeal approach. After controlled aspiration of cyst contents, the cyst can be delivered through the umbilical incision and extracorporeal cystectomy can be performed, which may improve traction/counter-traction and facilitate specimen handling while potentially reducing spillage. This “laparoscopic-assisted” concept has been described for large adnexal/ovarian cysts and for single-port transumbilical laparoscopic-assisted adnexal surgery [9, 23, 24]. Following extracorporeal steps, reinsertion of the port allows systematic inspection of the peritoneal cavity and meticulous irrigation, which is particularly important if any leakage is suspected. In our practice, a specialized multichannel single-port facilitates efficient switching between extracorporeal and intracorporeal procedures. Single-port techniques for adnexal surgery have been reported previously, including in pregnant patients; however, comparative evidence remains limited, and direct comparisons with laparotomy in a similar clinical setting are scarce [7, 8, 25–29]. 

Although estimated blood loss differed statistically between groups, the absolute volumes were low in both groups and no transfusions were required; thus, the clinical impact of this difference is likely modest and should be interpreted cautiously. Because pregnancy is a hypercoagulable state and CO2 pneumoperitoneum may contribute to venous stasis, venous thromboembolism (VTE) risk should be assessed and appropriate prophylaxis provided; current guidance supports mechanical prophylaxis (e.g., pneumatic compression devices) and early ambulation, with additional pharmacologic prophylaxis considered based on individual risk stratification [30–32]. The length of hospital stay in our cohort (approximately 4–8 days) was longer than that reported by some minimally invasive programs; this likely reflects local practice patterns for pregnant surgical patients, including more cautious inpatient observation for obstetric symptoms, pain control, and bowel function recovery, as well as institutional discharge workflows.

To our knowledge, the published evidence on laparoendoscopic single-site (single-port) adnexal surgery during pregnancy is still limited, consisting mainly of case reports and small case series. Accordingly, direct comparative data—particularly comparisons between a single-port assisted extracorporeal approach and laparotomy in a similar clinical setting—remain scarce.

Postoperative management regarding tocolysis remains debated. Previous reports have described heterogeneous practices, including short courses of postoperative tocolysis in selected patients; however, routine prophylactic tocolysis is not universally recommended. In our practice, prophylactic tocolysis is not routinely administered, and rescue tocolysis is considered only when signs or symptoms suggestive of preterm labor are suspected or documented, at the discretion of the obstetric team. In this cohort, only one patient in the laparotomy group received postoperative tocolytics because of uterine contractions, whereas no patients in the single-port group required postoperative tocolysis [31, 32]. 

Although the caesarean section rate was higher in Group 1 than in Group 2, this difference is likely driven by obstetric factors (e.g., prior caesarean delivery, nonreassuring fetal status, hypertensive disorders, maternal fever/infection) rather than the surgical approach, and residual confounding/selection bias is possible in this retrospective cohort. Importantly, neonatal outcomes among live births (birthweight, 5-minute Apgar score, and NICU admission) were comparable between groups [11, 13, 18]. 

Current guidance supports laparoscopic surgery during pregnancy when indicated and emphasizes multidisciplinary management to minimize maternal and fetal risks. Nevertheless, adverse pregnancy outcomes can occur and may be related to underlying obstetric conditions. In our study, one miscarriage and one stillbirth due to uterine rupture were observed in the laparotomy group; causality with the surgical approach cannot be inferred from this small retrospective cohort [30–32]. 

According to SAGES guidelines on laparoscopy during pregnancy, laparoscopy can be performed safely in any trimester when surgery is indicated. In published reports, gestational age at surgery has ranged from 4 to 31 + 4 weeks. Regarding first-trimester procedures, Lee et al. retrospectively reviewed 14 women with intrauterine pregnancies who underwent LESS for an adnexal mass; 11 were in the first trimester, and one experienced a miscarriage two weeks after surgery [29]. In another study by Jiang et al., 10 pregnant patients underwent LESS for gynecological acute abdomen in the first trimester; one patient who underwent LESS salpingectomy developed vaginal bleeding one week postoperatively and subsequently had a spontaneous abortion at 11 weeks’ gestation [33]. In our series, there was one preterm birth in the single-port group, one miscarriage in the laparotomy group, and one intrauterine fetal death.

This study has several limitations. First, the sample size was small, which limits statistical power, particularly for uncommon maternal and neonatal adverse events; therefore, the findings should be interpreted as hypothesis-generating rather than definitive. We did not perform an a priori power calculation because of the retrospective design and the limited number of eligible cases during the study period. Second, this was a single‑center retrospective cohort, and residual confounding and selection bias are possible despite comparable baseline characteristics. The small number of cases reflects the relative rarity of large/persistent ovarian cysts requiring surgery in early pregnancy, the fact that many cysts are treated before reaching a large size, and reduced referrals during COVID‑19 control measures [34], when more patients received care locally. Larger, multicenter prospective studies are needed to confirm these findings. Third, we did not adjust for potential confounders using multivariable models; therefore, observed associations may be influenced by unmeasured or residual confounding.

In conclusion, single-port assisted extracorporeal cystectomy appears to be a feasible option for selected pregnant patients with ovarian cysts ≥ 5 cm in the first and early second trimester. In this small retrospective cohort, it was associated with less blood loss and a shorter postoperative hospital stay, with comparable obstetric and neonatal outcomes. Given the non-randomized design and limited sample size, these findings should be interpreted cautiously and warrant confirmation in larger, multicenter studies.

## Data Availability

The datasets generated and analyzed during the current study are not publicly available due to patient privacy concerns but are available from the corresponding author on reasonable request.
